# Effect of Job Crafting Intervention Program on Harmonious Work Passion and Career Commitment among Nurses: A Randomized Controlled Trial

**DOI:** 10.1155/2023/9623866

**Published:** 2023-07-13

**Authors:** Heba E. El-Gazar, Shymaa Abdelhafez, Nashwa Ibrahim, Mona Shawer, Mohamed A. Zoromba

**Affiliations:** ^1^Nursing Administration Department, Faculty of Nursing, Port Said University, Port Said, Egypt; ^2^Psychiatric and Mental Health Nursing Department, Faculty of Nursing, Mansoura University, Mansoura, Egypt; ^3^Technical Institution of Nursing, Mansoura, Egypt; ^4^Nursing Department, College of Applied Medical Sciences, Prince Sattam Bin Abdulaziz University, Al-Kharj, Saudi Arabia; ^5^Faculty of Nursing, Mansoura University, Mansoura, Egypt

## Abstract

**Aim:**

This study aimed at evaluating the effects of a job crafting intervention program for nurses on their job crafting behaviors, harmonious work passion, and career commitment.

**Background:**

Nurses generally work in suboptimal environments with chronic low resources and high demands. Job crafting may be a cost-effective strategy to deal effectively with such environments. However, its effectiveness as a nursing intervention program remains unclear.

**Methods:**

An open-label randomized controlled trial was conducted at a hospital in Port Said, Egypt. The study enrolled 94 nurses. Participants were assigned randomly to the intervention group (*n* = 47) or the control group (*n* = 47). The intervention group had a 2-day workshop, 3 weeks of job crafting implementation, and a reflection session, whereas the control group participated in a 1-day workshop. Data were collected at baseline, 2 weeks, and 3 months after the intervention in both groups by using the Job Crafting Scale, Job Crafting Knowledge Questionnaire, Harmonious Work Passion Scale, and Career Commitment Scale.

**Results:**

Compared with the control group, the intervention group experienced a higher level of job crafting behaviors and reported a greater improvement in harmonious work passion, but not in career commitment.

**Conclusion:**

Nurses can be trained on job crafting behaviors, which can lead to the maximization of job resources, optimization of job demands, and enhancement of nurses' harmonious work passion. *Implications for Nursing Management*. Nursing managers should train nurses regularly on how to be job crafters because it is an effective tool that helps nurses deal with limited job resources and increased job demands and makes them more harmoniously passionate about their work.

## 1. Background

Nursing is a highly challenging profession [1]. Nurses worldwide are always under intense pressure [2, 3]. By their professional nature, they work in an environment with chronic high job demands and low resources [4]. In Egypt, nurses work in more complex conditions characterized by poor staffing, long shifts, extensive responsibilities, unclear job description, increased stress, high workload, and demotivation [5]. Working in such a suboptimal environment has adverse effects on nurses and places them in unfavorable circumstances [6]. To address these problems, nurses have proposed job crafting as a strategy to maximize these limited resources and optimize the increased job demands [7, 8]. Job crafting may be the solution for many problems nurses face in their workplace [9].

Wrzesniewski and Dutton initially presented the term “job crafting,” resulting from the physical and mental changes that people experience in a certain task or relationship boundary of their profession [10]. Recently, job crafting was viewed as a self-initiated behavior aimed at balancing between demands and resources of job [8]. Job crafting was operationalized from two dominant perspectives. The first is the Wrzesniewski and Dutton perspective, which theorizes that job crafting has three aspects, namely, task, relational, and cognitive crafting. Task crafting involves altering the scope or number of job tasks (e.g., considering taking an additional task as enjoyable). Relational crafting means improving the quality and/or the quantity of work relationships (e.g., less interaction with an unfavorable person). Cognitive crafting is changing the way people view their jobs (e.g., a nurses reframe their job from serving patients to saving patient's life) [10].

The second and most recent perspective is Tims et al.'s perspective, which is based on the Job Demands-Resources (JD-R) model. This study adopted this perspective. According to this perspective, job crafting is categorized into three aspects: increasing job resources, seeking challenge, and decreasing job demands [7]. Job resources are job elements that help in accomplishing work goals (e.g., colleagues' support, job autonomy, and supervisors' feedback). Challenging a job means accepting demands that are perceived as rewarding and worthy of one's effort (e.g., sharing of knowledge, participating in a certain committee, and mentoring nursing students). Job demands are job elements that need consistent physical, mental, and emotional efforts (e.g., time pressure and dealing with difficult patients) [8].

Practicing job crafting among nurses has become critical and can lead to multiple positive outcomes [11]. It is a cost-effective strategy by which nurses can improve their functioning at work [12]. Nurses who can craft their job are more able to balance between the needs of the organization and their preferences and abilities [13], to have higher levels of well-being, productivity, and meaningful work, and to give high-quality care [14]. Further, job crafting increases nurses' identity and autonomous internalization, which foster work passion [15]. One of the most important forms of work passion is harmonious work passion [16].

Harmonious work passion is a new concept in organizational behavior that has gained increased attention in the recent nursing management literature [17]. It is related to the autonomous internalization of one's activity, allowing one to perform such an activity freely and willingly [16]. Notably, work passion is categorized into two types: harmonious work passion and obsessive work passion [18]. In this study, we adopt harmonious work passion as it represents the former type of passion, characterized by individuals' identity towards an activity and their internal motivation for doing it [17], making it a suitable target for the study. Studying factors that foster nurses' harmonious passion has become crucial because this type of passion has tremendous benefits [19]. For example, harmoniously passionate nurses are highly intrinsically motivated to seek more information [17], are more satisfied with their work, are less susceptible to conflict, are less prone to burnout [20], engage more in their work, and experience less exhaustion and job stress [21].

Nurses are the main source of healthcare services in health institutions [22]. They are responsible for patient care and treatment against disease and mortality [1]. Thus, enhancing nurses' career commitment is necessary [23]. Career commitment is described as individuals' identification with and attachment to their profession [24]. Nurses with a high level of career commitment are more likely to show favorable work consequences, including higher citizenship behaviors and organizational commitment and decreased deviant behaviors and intentions to quit their profession [25].

Generally, nurses have a unique role in modern medicine [26]. However, nurses' poor work conditions have deleterious consequences, putting the safekeeping and quality-of-care standards at risk [27]. Therefore, nursing scholars become more interested in studying job crafting [11], and others recommend interventions to foster it among nurses because of the urgent concern and the belief that it is a powerful strategy that can change their work environment positively [13]. Job crafting training empowers nurses to have greater control over their work, build a motivating work climate, maximize job resources, optimize increased job demands, enhance cognitive and behavioral attitudes towards change, and reduce exhaustion [28]. Additionally, it has been shown to enhance work engagement [29] and self-efficacy [30]. These benefits underscore the importance of providing job crafting training for nurses. However, job crafting studies in nursing are still few [31], and among them, only one focused on job crafting intervention [12]. Moreover, in the non-nursing field, studies that included job crafting intervention yielded contradictory results, and job crafting as a trainable behavior remains uncertain [32]. Therefore, to address these gaps, this study primarily aimed at testing if job crafting intervention can stimulate job crafting behaviors among nurses.

Training nurses on how to craft their job may lead to many positive outcomes. Job crafting training previously improved nurses' well-being and performance [12]. However, researchers found no published studies investigating the effect of job crafting intervention program on harmonious work passion or career commitment in nursing or non-nursing field, despite the critical need of studying factors that can increase nurses' harmonious work passion [19] and career commitment [33]. Hence, the secondary objective of the present study was to fill these gaps by examining the effect of job crafting intervention program on nurses' harmonious work passion and career commitment.

## 2. Theoretical Framework

Job crafting intervention is a training program designed to encourage individuals to proactively redesign their jobs by optimizing available job resources, managing increased job demands, and adapting to new job challenges [34]. Job crafting intervention is based on two main approaches: (1) the Wrzesniewski and Dutton approach, which frames job crafting as proactive changes in task, relational, and cognitive job aspects [10], and (2) the Tims et al. approach, which is based on the Job Demands-Resources (JD-R) theory [7].

The JD-R theory postulates that individuals require a sufficient amount of job resources to effectively cope with the demands they encounter in their work. According to this theory, organizations play a crucial role in providing optimal job demands and resources to their employees. However, individuals also possess the autonomy to proactively modify their job demands and resources, thereby contributing to a better alignment between individuals and their jobs. Consequently, this leads to enhanced optimal functioning within the workplace [35]. Job crafting, when framed within this context, refers to the ability of individuals to change three types of job characteristics: job resources, seeking challenge, and job demands [7].

Previous studies have explored the outcomes of job crafting intervention and demonstrated its positive effect on enhancing work engagement, job performance [29], career satisfaction [36], and well-being [12]. Job crafting intervention increases individuals' awareness of their own preferences and needs, empowering them to take action to make changes. The job crafting intervention provides individuals with specific techniques and tools they can use to identify areas where they can make changes to manage their job resources and demands. Moreover, job crafting intervention encourages them to formulate a job crafting plan, which increases their adherence to job crafting behaviors [37]. Hence, job crafting intervention leads to an increase in job crafting behaviors and all related dimensions. Therefore, the study hypothesized that

  H1. Nurses participating in the job crafting intervention will exhibit higher levels of job crafting behaviors and all related dimensions after the intervention compared with those in the control group.

### 2.1. Job Crafting Intervention and Harmonious Work Passion

Harmonious work passion is an essential factor that has significant implications for the well-being and performance of nurses [38]. However, nurses can lose their harmonious passion due to the intense physical, emotional, relational, and social pressures they face while practicing their profession [39]. Additionally, a lack of control over their work environment can also lead to reduced passion [40]. Training nurses on how to craft their job can be an effective strategy to help them manage physical, emotional, relational, and social pressures and gain control over their work environment [10]. Hence, training nurses on how to craft their job can enhance their passion towards their work. Therefore, the study hypothesized that:

  H2. Nurses participating in the job crafting intervention will experience a higher level of harmonious work passion after the intervention compared with those in the control group.

### 2.2. Job Crafting Intervention and Career Commitment

Career commitment is achieved when nurses are able to align their career path with their personal preferences and interests [25]. Job crafting training equips individuals with the necessary tools to proactively shape their work environment to better fit their skills, values, and interests [41]. Additionally, practicing job crafting and gaining increased control over their work can foster a sense of ownership and investment in their careers [42]. Furthermore, job crafting training can enhance nurses' job engagement and well-being [12], which can further contribute to their career commitment [43]. Hence, job crafting training can improve career commitment among nurses. Therefore, the study hypothesized that

  H3. Nurses participating in the job crafting intervention will have a higher level of career commitment after the intervention compared with those in the control group.

## 3. Subjects and Methods

### 3.1. Study Design

This study was a two-arm, parallel, open-label randomized controlled trial (RCT) that conforms to the Consolidated Standards of Reporting Trials (CONSORT) guidelines [44]. The study protocol was registered at ClinicalTrials.gov (Identifier code: NCT05329805; 15/04/2022).

### 3.2. Participants and Setting

**Figure 1:**
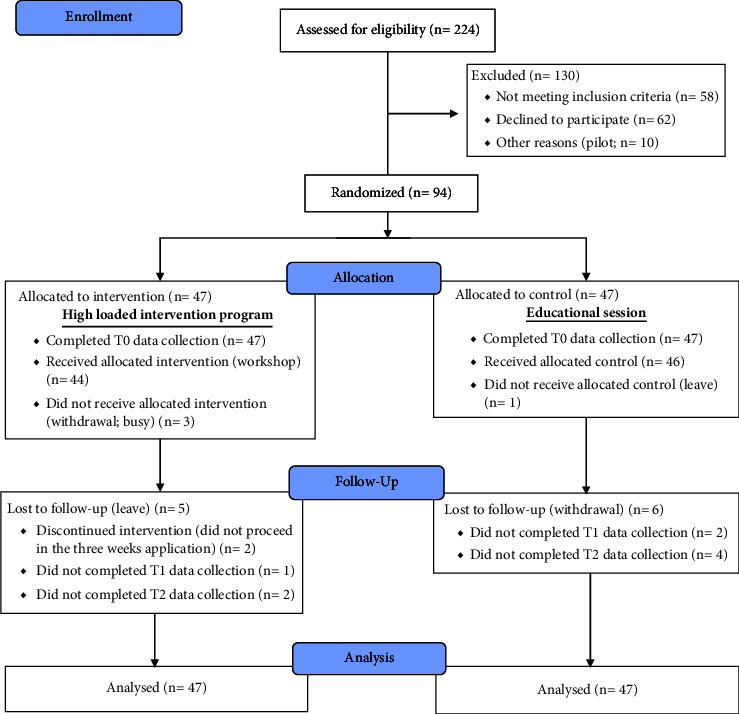
Consort flow diagram.

This study recruited nurses working 12 hours per shift for 186–195 hours a month at one of the universal health insurance hospitals in Port Said Governorate, Egypt. Nurses who were licensed staff nurses, worked in a ward, and had at least 6 months of experience were included in the analysis. In contrast, nurses who were involved in any other intervention program within the last 12 months, working in clinics, or holding an administrative position were excluded. Of the 224 invited participants, 58 did not meet the inclusion criteria, 62 declined to participate, and 10 were piloted. Ultimately, 94 were included and randomly allocated to the intervention group (*n* = 47) or the control group (*n* = 47). Overall, 79 (39 in the intervention group and 40 in the control group) completed the intervention up to T2 data collection. The attrition rate from the baseline (T0) until T2 data collection was 15.95%.  Figure 1 illustrates the consort flow diagram of the participants.

### 3.3. Sample Size Calculation

A prior sample size was estimated using the G^*∗*^Power 3.1.9.7 Software [45] for repeated measures analysis of variance (ANOVA) design (within-between interactions) with effect sizes of 0.26 and was obtained from the meta-analysis on job crafting intervention that included healthcare professionals [29] (*α* error = 0.001, power = 0.95). A sample of 72 participants (36 for each group) was estimated. Considering the possibility of a 30% attrition rate resulting from high dropout/loss to follow-up among Egyptian participants [46], the recruitment target was 94 participants (47 in each group).

### 3.4. Randomization and Blinding

An independent researcher randomly assigned the participants to the intervention or control group by using the “Research Randomizer” web-based program [47]. The number of groups needed and the number of potential participants were input into the program, which generated two sets of 94 unique, sorted numbers arranged from the least to the greatest and allocated randomly to either Group 1 (representing the control group) or Group 2 (representing the intervention group) in a 1 : 1 ratio. The allocation sequence was concealed by using opaque sealed envelopes.

Blinding was not feasible in this study because the intervention provided was an educational program, which made the blinding of the researcher or the participants to the group allocation difficult [48]. Nevertheless, to minimize the risk of bias, the data collection and analysis were performed by authors (SM, MS, and MZ) were not involved in providing the intervention program conducted.

### 3.5. Intervention

The job crafting intervention program was developed according to the Michigan Job Crafting Exercise [37] and was operationalized on the basis of the JD-R theory principles [49]. The intervention aimed to train the participants in maximizing their job resources, optimizing the increased job demands, and adapting new job challenges, which may be useful in enhancing their harmonious work passion and career commitment.

Prior to the intervention program, potential participants and nursing managers with different levels were interviewed to understand the work context, which included the following: what does good performance mean from their point of view, what hinders them from doing so, what helps them to provide high-quality care, what tasks represent challenges, and why they did not assume such challenges. This information was utilized to prepare a tailored intervention and customized examples in creating the intervention.

Five-expert committee included two nursing administration professors, one nursing manager, and two staff nurses with master's degrees were asked for suggestions on the content and structure of the intervention materials. The intervention materials were modified based on their suggestions and sent back to the committee for approval. Then the intervention materials were pilot tested to ensure their quality and clarity.

Thereafter, the intervention was conducted, consisting of a 2-day workshop, 3 weeks of job crafting implementation, and a reflection session. The workshop consisted of four 60–90-minute sessions with 2 sessions per day and 30 min rest between sessions over 2 days. In session 1, the researcher introduced the theoretical background. In session 2, the JD-R model was discussed in detail, and the participants were requested to share their personal job crafting experiences. In session 3, they were invited to participate in the Michigan Job Crafting Exercise [37], which included job analysis, personal analysis, and job-personal analysis. As a result of completing the exercise, each participant was expected to be aware of the following: resources that could be increased, the idea that demands could be decreased without interrupting the work, and areas that represent a challenge to them. In the fourth session, the participants were invited to prepare their own “Personal Crafting Plan.” Table 1 details the content of the workshop.

At the end of the workshop, each participant received a booklet of the teaching material, which was developed according to an intensive literature review [8, 10, 37, 49–51] and the nurses' needs that were identified through interviews.

Furthermore, a list of job crafting activities was distributed. This list was developed by the researchers according to a previous job crafting study [31, 32, 52] and input from interviews. This list contained four sections. The first section included a set of points to increase job resources, such as “Asking for help from your colleague or your leaders if you need it” and “Clean your desk to work more efficiently.” The second section included a set of points to increase challenging job demands, such as “Offer to be responsible for making the nursing schedule in a certain month” and “Share your head nurse in writing certain documents e.g., lab-ray or sterilization.” The third section includes a set of points to decrease hindering demands, such as “Checking emails only at certain times during the day” and “Encourage patient to perform daily morning care by himself.” The fourth section included five potential nursing situations with suggested job crafting activities. Each situation was accompanied by activities that can increase resources, increase challenging demands, and decrease hindering demands. Giving a list of job crafting activities makes it easier for nurses to practice job crafting [32].

After finishing the workshop, the participants were asked to engage in job crafting activities for 3 weeks guided by the job crafting activity list and the plan developed by the researchers. The participants were then asked to target activities related to increasing resources, optimizing job demands, and seeking challenges in weeks 1, 2, and 3, respectively. During this 3-week intervention period, the participants were invited to a WhatsApp group, which was formed to encourage, guide, share experiences, and send reminders twice weekly to engage in the assigned behaviors.

After completing the 3-week intervention program, a reflection session was held. In this session, participants were asked to evaluate their success, obstacles, solutions, and future plans for how they can craft their job. At the end of the session, the researcher expressed appreciation to each group member.

### 3.6. Control Group

In the control group, the participants received only the content provided in the first and second sessions. They neither participate in the Michigan Job Crafting Exercise nor prepare the Personal Crafting Plan. Unlike the intervention group, the control group was not required to participate in the 3-week job crafting intervention or the reflection session.

### 3.7. Measures

The study measures included scales that measure job crafting behaviors, knowledge about job crafting, harmonious work passion, career commitment, and the demographic information form. The researchers translated the scales, which were initially developed in English, into Arabic using the committee approach to translation [53]. The committee included four members who translated the scales independently and in parallel. These four members included 2 Egyptian nursing academics, 1 nurse who was proficient in English and familiar with the healthcare environments, and 1 English educator who was an Arabic native and fluent in English. All committee members approved the final version of each scale. All these scales were self-reported and completed by both group participants across the three time points.

#### 3.7.1. Primary Outcome

*(1) Job Crafting Scale*. The Job Crafting Scale [8], which has 13 items, was used to assess the level of nurses' job crafting behaviors. It includes three dimensions: seeking resources (6 items), seeking challenges (3 items), and reducing demands (4 items). A sample item includes “I make sure that my work is mentally less intense.” The scale answers range from 1 (never) to 5 (always). Higher scores indicate higher levels of nurses' job crafting behaviors. In its original version, the Cronbach's alpha for scale dimensions is 0.69–0.76. In our study, its reliability across the three time points was acceptable (*α* = 0.799–0.901).

#### 3.7.2. Secondary Outcomes

*(1) Job Crafting Knowledge Questionnaire*. This questionnaire is a 26-item multiple-choice questionnaire that was used to assess the level of nurses' knowledge of job crafting. It was developed by the researchers according to previous studies [8, 10, 49, 51]. The 26 questions particularly assessed nurses' understanding of the job crafting concept (1 question), the importance of job crafting (1 question), characteristics of job crafting (2 questions), models (8 questions), and JD-R theory and its application strategy (14 questions). The score ranges from 0 to 26, with higher scores indicating greater knowledge.

*(2) Harmonious Work Passion Scale*. The Harmonious Work Passion Scale, which has 7 items, was created by Vallerand et al. to assess nurses' harmonious passion toward their work [18]. A sample item includes “This work allows me to live memorable experiences.” Responses were based on a 7-point Likert scale, with 1 representing “Not agree at all” and 7 representing “Very strongly agree.” Higher scores indicate greater harmonious work passion. The Cronbach's alpha in its original version was 0.79. In this study, the reliability across the three time points was acceptable (*α* = 0.711–0.894).

*(3) Career Commitment Scale*. The Career Commitment Scale [24], which consists of 8 items, was specifically designed to assess career commitment among nurses. The scale has three negatively worded items that are scored in reverse. A sample item includes “I definitely want a career for myself in nursing.” A 5-point Likert scale, ranging from 1 (strongly disagree) to 5 (strongly agree), was used. Cronbach's alpha in its original version was 0.85. In this study, the reliability across the three time points was acceptable (*α* = 0.736–0.753).

#### 3.7.3. Demographic Information Form

This form consists of questions related to age, gender, marital status, education, nursing tenure, and status of receiving job crafting training before.

### 3.8. Data Collection and Procedure

**Figure 2:**
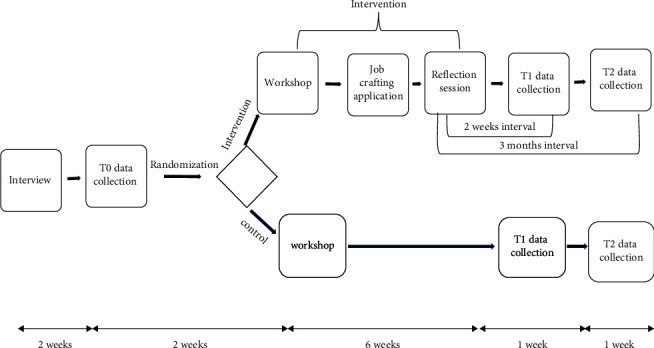
Graphical representation of the study design.

Data were collected from the beginning of February 2022 to July 2022 in a longitudinal setup with a three-wave measurement (i.e., T0 = the baseline measurement collected 2 weeks before the intervention, T1 = the second measurement collected 2 weeks after the intervention, and T2 = the third measurement collected 3 months after the intervention (Figure 2)). Initially, permission to implement the study was obtained from the hospital administrators. Participants were recruited through announcements in their hospitals. The study purposes and procedures were thoroughly explained to all potential participants. Nurses who met the inclusion criteria and agreed to participate were asked to sign an informed consent form. After completion of the baseline measurement (T0), the nurses were randomly assigned to either the intervention or the control group. Three workshops were held with a maximum of 16 nurses per workshop. These workshops were conducted in the hospital education room.

To ensure consistent and effective implementation, a detailed implementation plan was developed, which outlined the specific activities, resources, and timelines for the intervention. Evidence-based teaching strategies were adhered to during program delivery, which was provided by the first author, who holds a PhD in Nursing Management and has extensive teaching experience. In addition, validated intervention materials, including booklets, brochures, and a list of job crafting activities, were developed and utilized.

To ensure participant retention, the program was scheduled at a convenient time for the study participants. In addition, multiple ways of maintaining contact with participants (i.e., personal phone contact, WhatsApp group, and email) were utilized to remind them to attend sessions and engage in the assigned job crafting behaviors.

### 3.9. Validity

The intervention program and data collection instruments used in this study were validated by 5 experts working in clinical and academic settings.

### 3.10. Pilot Study

A pilot study was conducted to assess the clarity, feasibility, validity, and reliability of the study measures, the amount of time taken for data collection, and the quality and clarity of the intervention materials. Ten nurses who were not included in the study sample but met the inclusion and exclusion criteria participated in the pilot study. Results revealed that the study measures and intervention materials were understandable and that no modifications were required.

### 3.11. Data Analysis

Data were analyzed using the Statistical Package for Social Sciences version 24.0 (IBM Corp, Armonk, NY, USA). Normally distributed data were evaluated using the Shapiro–Wilk test. The participants' characteristics were summarized using descriptive statistics. The homogeneity of the intervention and control groups' baseline characteristics was tested using the independent samples *t*‐tests for the continuous data and the chi‐squared (*χ*^2^) test for the categorical data.

The study used intention-to-treat analyses [54] on participants who completed one data point (i.e., T0) at least (*n* = 94). The missing data (*n* = 15) were compensated by the “last observation carried forward” (LOCF) method [55]. Changes in the study outcomes within groups (over time: T0, T1, and T2) and between groups (intervention and control groups) were analyzed by a two-way repeated-measures ANOVA. Pairwise comparisons to further assess the significance between groups were conducted using Bonferroni correction to guard against type I errors.

The effect size for the intervention was calculated using the partial eta-squared (*η*_*p*_^2^) for differences indicated to be statistically significant and interpreted as small (0.01–0.06), medium (0.06–0.14), and large (≥0.14) effects [56]. All statistics were two-sided, and the significance level was set at <0.05.

#### 3.11.1. Ethics Consideration

The Research Ethics Committee of the Faculty of Nursing in the Mansoura University, Egypt, approved the study protocol (NO: P.0231). The study conformed to the principles of the Declaration of Helsinki. The participants were explained the study objective and protocol, and only those who provided informed consent were included. Voluntary participation, autonomy, and confidentiality of the information gathered were confirmed.

## 4. Results

Most of the participants were female in both groups (intervention: 85.1%, control: 78.7%), with a mean age of 32.74 ± 6.18 and 34.29 ± 6.69 years in the intervention and control groups, respectively. Most of the participants in the intervention group were unmarried (55.3%) and held a diploma degree (31.9%), with a mean nursing tenure of 11.47 ± 5.86 years. In the control group, most of the participants were married (59.6%) and held an associate degree (44.7%), with a mean nursing tenure of 12.47 ± 6.93 years. Overall, the baseline characteristics were not significantly different between the two groups (*p* > 0.05), indicating that these characteristics were well balanced between these groups (Table 2).

### 4.1. Primary Outcome

#### 4.1.1. Job Crafting Behaviors

**Figure 3:**
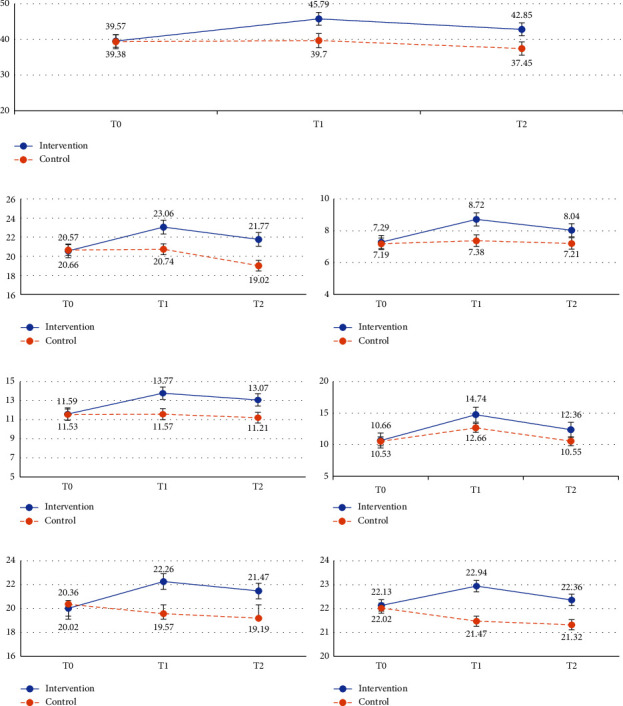
Mean changes of the primary and secondary outcomes. (a) Total job crafting, (b) seeking resources, (c) seeking challenges, (d) reducing demands, (e) job crafting knowledge, (f) harmonious work passion, and (g) career commitment.

As shown in Figure 3 and Table 3, the total scores of job crafting behaviors and their dimensions (seeking resources, seeking challenges, and reducing demands) were not significantly different between the intervention and control groups at baseline (*p* > 0.05).

Regarding the total score of job crafting behaviors, the repeated measures ANOVA showed that the interaction effect of group × time was significant (*F* (2, 165) = 33.78, *p* < 0.001), with a large effect size (*η*_*p*_^2^ = 0.27). Furthermore, the pairwise comparison revealed that the intervention group had shown higher job crafting behaviors than the control group at T1 (M ± SD = 45.79 ± 5.19 vs. 39.70 ± 5.59, *p* < 0.001) and T2 data points (42.85 ± 5.01 vs. 37.39 ± 5.38, *p* < 0.001). In terms of time, the mean of job crafting behaviors of the intervention group was increased by 6.22 in T1 compared with that in T0 data collection; meanwhile, the result was nearly similar in the control group (difference = 0.32). However, the total score of job crafting behaviors declined to −2.94 in T2 compared with that in T1 in the intervention group and to −2.25 in the control group, but in the intervention group, it was still higher than T0 (3.28) (Figure 3, Table 3).

Concerning job crafting dimensions, the results showed a significant group × time interaction effect for seeking resources (*F* (2, 184) = 15.45, *p* < 0.001), seeking challenges (*F* (2, 184) = 8.91, *p* < 0.001), and reducing demands (*F* (2, 144) = 14.17, *p* < 0.001). However, the main effect of groups on seeking challenges was not significant (*F* (1, 92) = 3.58, *p* = 0.062), revealing that after excluding the group effect, the level of seeking challenges was still significant over time. Moreover, the effect size was medium for seeking resources, seeking challenges, and reducing demand (*η*_*p*_^2^ = 0.14, 0.09, and 0.13, respectively; Figure 3, Table 3). Thus, H1 was supported.

### 4.2. Secondary Outcomes

#### 4.2.1. Job Crafting Knowledge

At baseline, job crafting knowledge was not significantly different between the two groups (*p* = 0.837). The results indicated a significant interaction between the group and time factors (*F* (2, 167) = 10.29, *p* < 0.001), with a medium effect size (*η*_*p*_^2^ = 0.10). Both groups showed increased knowledge in the T1 data point compared with that in the T0 data point, but the intervention group gained more knowledge than the control group by a mean difference of 2.08 (*p* = 0.011). In the T2 data point, the knowledge of both groups decreased compared with that in the T1 data point, and it was similar to T0 in the control group, but in the intervention group, it was still higher than T0 by 1.7.

#### 4.2.2. Harmonious Work Passion

At baseline, harmonious work passion showed no significant differences between the two groups (*p* = 0.663). The interaction effect of group × time was significant (*F* (2, 160) = 14.95, *p* < 0.001, *η*_*p*_^2^ = 0.14). The intervention group gained higher harmonious work passion than the control group at T1 (22.26 ± 4.68 vs. 19.57 ± 3.31, *p* = 0.002) and T2 data points (21.47 ± 4.12 vs. 19.19 ± 2.68, *p* < 0.002). In time comparison, the mean of harmonious work passion in the intervention group improved by 2.24 from baseline to T1 and by 1.45 from baseline to T2. Likewise, the harmonious work passion scores in the control group slightly declined over time (T0: 20.36 ± 3.00, T1: 19.57 ± 3.31, and T2: 19.19 ± 2.68; Figure 3, Table 3). Thus, H2 was supported.

#### 4.2.3. Career Commitment

As shown in Figure 3 and Table 3, career commitment was not significantly different between the two groups at baseline (*p* = 0.892). The interaction effect of group × time was also not significant (*F* (2, 184) = 2.89, *p* = 0.058). Likewise, the main effects of group (*F* (1, 92) = 1.76, *p* = 0.188) and time (*F* (2, 184) = 0.803, *p* = 0.450) showed no significance. Consequently, H3 was rejected.

## 5. Discussion

Given that nurses constantly face high demands and few resources, which have negative outcomes on them [23], they must be trained on strategies that help them deal with such challenges, and the effectiveness of these strategies should be evaluated. Therefore, this study aimed at evaluating the effects of implementing a job crafting intervention program for nurses on their job crafting behaviors, harmonious work passion, and career commitment.

Results showed that nurses who underwent the program (the intervention group) reported significantly higher levels of job crafting behaviors than those who did not (the control group) immediately and 3 months after the intervention, with a large effect size. This finding is valuable because it indicates that job crafting is a trainable behavior. These results are in line with earlier studies showing that job crafting behavior could be fostered through intervention [57, 58]. However, these results are contradictory with a quasi-experimental study conducted among Dutch healthcare providers; in this previous study, the job crafting training did not improve job crafting behaviors [59]. The discrepancy in results may be caused by the difference in work environment. Kuijpers et al. attributed their program's ineffectiveness to contextual factors such as poor leadership and organizational climate [59], whereas the present study was conducted in a universal health insurance hospital that has obtained accreditation and has a culture that reinforces such changes.

For job crafting dimensions, the intervention was effective in increasing seeking resources and decreasing job demands in the intervention group compared with the control group over time. However, for the dimension of seeking challenges, the intervention succeeded in enhancing seeking challenges in the intervention group compared with the control group immediately after the program, but during follow-up, the intervention group failed to maintain such behaviors. This result suggests that to maintain challenging behaviors among nurses, more reinforcement and ongoing support may be needed. Supporting this notion, Dubbelt et al. stated that seeking challenges is a less motivating behavior in an environment with high demands [36]. These results were partially supported by Gordon et al., who revealed that after offering job crafting training to nurses, the intervention group showed higher levels of seeking resources and decreasing job demands but not for seeking challenges [12].

Furthermore, job crafting knowledge increased in both groups, but the intervention group gained more knowledge than the control group. Despite the fact that nurses' knowledge in the control group was also increased, it was not enough to motivate them to adhere to job crafting activities. This result may be explained by the idea that the intervention activities performed by nurses in the intervention group after the workshop enhanced their intention and ability to practice job crafting. Interestingly, these results could help hospital managers design guidelines and programs to increase nurses' job crafting behaviors, considering that workshops and lectures are not sufficient for increasing such behaviors in nurses and should be accompanied with intervention activities.

Especially the job crafting intervention succeeded in improving nurses' harmonious passion toward their work. As anticipated, the intervention group gained a higher harmonious work passion than the control group over time. This result could be explained by the idea that job crafting behaviors enable nurses to create a more fitting and controllable working environment [60], which enhances nurses' feelings of self-efficacy and gets rid of a sense of powerlessness [61]. These positive outcomes may motivate nurses to invest their time and energy and experience more passion at work. Unfortunately, no study has examined yet the effect of job crafting interventions on harmonious work passion. Nonetheless, these findings are consistent with previous results obtained from an observational study that reported a positive relationship between job crafting and harmonious work passion [62].

Notably, the increased levels of job crafting behaviors, knowledge, and harmonious work passion among nurses in the intervention group began to decrease after 3 months of finishing the intervention. Therefore, nursing managers and hospital administrators should regularly organize job crafting training for nurses.

Contrary to our expectations, the intervention was not effective in enhancing nurses' career commitment. The reason may be that career commitment arises from the accumulation of perceived experiences [63]. Hence, the researchers suggest combining upcoming job crafting interventions with other activities, such as mindfulness, guided imagination, relaxation, and stress management activities. Another reason is the idea that the effect of the job crafting intervention on outcomes that go beyond one's job (i.e., career) is a sleeper effect, which needs more time to manifest. Thus, the intervention may work first by improving job engagement and satisfaction, and the effect on career commitment could be realized later in time. Despite being the first study to evaluate the effect of a job crafting intervention program on career commitment, the present findings are in line with those of Dubbelt et al., who implemented a job crafting program and reported that the effect of intervention on career satisfaction was not significant [36]. However, these results are contrary to the descriptive study of Chang et al., who showed that job crafting has a direct effect on career commitment [64].

### 5.1. Limitations

One of the limitations of this study is that the participants of the intervention and control groups were enrolled from the same hospital and potentially from the same units, possibly leading to cross-contamination. The study was conducted in one hospital to ensure that all study participants were subject to the same conditions, including working environment, serving the same patient populations, nursing practices, and workload. This approach aimed to increase the homogeneity of the study sample and reduce the potential for confounding factors that could affect the results of the study.

To avoid cross-contamination risk, the intervention group participants were asked not to share any information related to the intervention with others until study completion. To reinforce this message, regular reminders were sent to the participants via WhatsApp and e-mail. After completing the study, the control group participants were asked whether they had received any information about the job crafting activity during the study, and they confirmed that they had not. Another limitation is that the study lacked a guided website intervention. Nonetheless, a WhatsApp group was used to encourage, guide, and remind nurses; this application may be an alternative to the website. In addition, the study did not explore the mechanisms through which job crafting intervention works. Such mechanisms should be explored in future studies.

### 5.2. Implications

The study presents several meaningful practical implications for frontline nurses and nurse managers. The findings of this study provided evidence that nurse managers could promote nurses' job crafting behaviors by implementing tailored job crafting intervention programs. The study also highlighted the favorable outcomes that nurses and their managers can gain from the job crafting intervention. The job crafting intervention can be used as a promising method to help nurses cope with limited job resources and increased job demands and make them more harmoniously passionate about their work; thus, this study urges nursing managers to offer more opportunities for job crafting among nurses.

Moreover, the study findings guide nurse managers in designing job crafting interventions. This study suggests combining theoretical lectures with the intervention activity because workshops and lectures alone are insufficient to improve nurses' job crafting behaviors. In addition, to maximize the potential benefits, the study recommends adding other activities, such as mindfulness and stress management activities, to the job crafting activities. Nurses should also be trained on job crafting regularly.

Furthermore, the study yielded some theoretical implications. The current study contributes to job crafting literature and Job Demands-Resources (JD–R) theory in several ways. It provided evidence that job crafting is a trainable behavior within the nursing context. It also assured that job crafting interventions based on the Job Demands-Resources (JD–R) theory is effective in fostering harmonious work passion for nurses. However, the study results did not observe that the job crafting intervention has any effect on boosting nurses' career commitment. Further intervention studies are needed to confirm these results.

## 6. Conclusions

This RCT showed that in the context of the Job Demands-Resources (JD–R) theory, the job crafting intervention, which involves workshops and job crafting activities, proved to be effective in enhancing nurses' job crafting behaviors and harmonious work passion but not their career commitment. Further evidence is needed.

## Figures and Tables

**Table 1:** Job crafting training sessions, goals, and activities.

Sessions	Goals	Program activities
First session (theoretical background)	(1) Establish a group	(i) Warm-up game and acquaintances
	(2) Introduce the program	(ii) Establishment of a group contract
	(3) Introduce the concepts of job crafting	(iii) Clarification of the program's aim, activities, and timeline
	(4) Distinguish between job crafting and the traditional job design	(iv) Explanation of the concepts of job crafting by the researcher
	(5) Explore the importance of being a job crafter	(v) Group discussion about the difference between job crafting and the traditional job design
	(6) Identify the job crafting characteristics	(vi) Description of the importance of being a job crafter
	(7) Discuss different job crafting models	(vii) Demonstration of job crafting characteristics
		(viii) Explanation of the different job crafting models by the researcher
		(ix) Sharing of real-life examples in practicing each model by the participants

Second session (JD-R model)	(1) Focus on the job demands-Resources (JD-R) model	(i) Lecture on JD-R model
		(ii) Provision of examples of job resources, demands, and challenges related to nursing career
	(2) Exchange concrete experiences	(iii) Sharing of personal job crafting experiences in terms of maximizing resources, optimizing job demands, and seeking challenges with each other by the participants
		(iv) Review of the day
		(v) Provision of homework that aims to identify job resources, demands, and challenges other than those mentioned in the session

Third session (Michigan job crafting exercise)	(1) Complete the Michigan job crafting exercise in terms of the following:	(i) Sharing homework from the previous session
	(2) Job analysis	(ii) Brain storming about job resources they have in their workplace
	(3) Personal analysis	(iii) The group listed their job demands
	(4) Job-personal analysis	(iv) Group discussion about tasks which represent job challenges
		(v) Participants summarize individually their own strengths, motive, weakness point and obstacles they experience in their work and matched it with the task they performed

Fourth session (personal crafting plan)	(1) Create the personal crafting plan	(i) Participants individually formulating their own job crafting goals and activities they could practice to maximize job resources, adapt new challenges and optimize job demands to carry out in the upcoming three weeks
	(2) Clarify the next intervention	(ii) Discussion about the upcoming application
		(iii) Concluding all sessions

**Table 2:** Participant characteristics at baseline in the intervention and the control groups.

Characteristics		Total (*N* = 94) *N* (%)	Intervention group (*n* = 47) *n* (%)	Control group (*n* = 47) *n* (%)	*P*
Age (years)	Mean ± SD	33.52 ± 6.46	32.74 ± 6.18	34.29 ± 6.69	0.246^a^

Gender	Male	17 (18.1)	7 (14.9)	10 (21.3)	0.593
	Female	77 (81.9)	40 (85.1)	37 (78.7)	

Marital status	Married	49 (52.1)	21 (44.7)	28 (59.6)	0.215
	Unmarried	45 (47.9)	26 (55.3)	19 (40.4)	

Education	Diploma	26 (27.7)	15 (31.9)	11 (23.4)	0.748
	Associate	35 (37.2)	14 (29.8)	21 (44.7)	
	Bachelor	23 (24.5)	14 (29.8)	9 (19.1)	
	Postgraduate	10 (10.6)	4 (8.5)	6 (12.8)	

Nursing tenure (years)	Mean ± SD	11.97 ± 6.40	11.47 ± 5.86	12.47 ± 6.93	0.452^a^

SD, standard deviation. *Note*. All participants in both groups reported they ever received any training on job crafting before. ^a^Analyzed by *t*-test while others by *χ*-square cross-tab analysis.

**Table 3:** Change in study outcomes by the intervention and control groups overtime.

Outcomes	Time point	Intervention group (*n* = 47)	Control group (*n* = 47)	Pairwise comparison (*p*)	Group *F* (*p*)	Time *F* (*p*)	Group × time	
		Mean ± SD	Mean ± SD				*F* (*p*)	*η*_*p*_^2^
Total job crafting behaviors	T0	39.57 ± 5.99	39.38 ± 5.14	0.868	14.67 (<0.001)	38.68 (<0.001)	33.78 (<0.001)	0.27
	T1	45.79 ± 5.19	39.70 ± 5.59	<0.001				
	T2	42.85 ± 5.01	37.45 ± 5.38	<0.001				

Seeking resources	T0	20.57 ± 3.33	20.66 ± 3.24	0.900	6.49 (0.012)	17.65 (<0.001)	15.45 (<0.001)	0.14
	T1	23.06 ± 3.99	20.74 ± 3.41	0.003				
	T2	21.77 ± 3.58	19.02 ± 3.46	<0.001				

Seeking challenges	T0	7.29 ± 2.21	7.19 ± 2.35	0.821	3.58 (0.062)	15.17 (<0.001)	8.91 (<0.001)	0.09
	T1	8.72 ± 1.99	7.38 ± 2.03	0.002				
	T2	8.04 ± 2.06	7.21 ± 1.99	0.051				

Reducing demands	T0	11.59 ± 2.79	11.53 ± 1.86	0.897	10.75 (0.001)	13.38 (<0.001)	14.17 (<0.001)	0.13
	T1	13.77 ± 2.42	11.57 ± 2.47	<0.001				
	T2	13.04 ± 2.44	11.21 ± 1.93	<0.001				

Job crafting knowledge	T0	10.66 ± 2.82	10.53 ± 3.17	0.837	4.07 (0.047)	94.29 (<0.001)	10.29 (<0.001)	0.10
	T1	14.74 ± 3.82	12.66 ± 3.96	0.011				
	T2	12.36 ± 3.29	10.55 ± 3.65	0.013				

Harmonious work passion	T0	20.02 ± 4.41	20.36 ± 3.00	0.663	4.87 (0.030)	3.28 (0.047)	14.95 (<0.001)	0.14
	T1	22.26 ± 4.68	19.57 ± 3.31	0.002				
	T2	21.47 ± 4.12	19.19 ± 2.68	0.002				

Career commitment	T0	22.13 ± 4.17	22.02 ± 3.37	0.892	1.76 (0.188)	0.80 (0.450)	2.89 (0.058)	—
	T1	22.94 ± 3.57	21.47 ± 3.62	0.051				
	T2	22.36 ± 3.37	21.32 ± 3.28	0.132				

SD, standard deviation; T0, baseline; T1, immediate postintervention; T2, 3-month follow-up.

## Data Availability

The datasets generated during and analyzed during the study are available upon reasonable request from the corresponding author.
